# Immediate changes in self-reported apprehension and uncertainty across 41 emergency scenarios following a structured emergency medicine course

**DOI:** 10.3389/fmed.2026.1943532

**Published:** 2026-10-29

**Authors:** Niels-Benjamin Adams, Hendrik Drinhaus, Martin Strothe, Marc J. Schieren, Kathrin Möllenhoff, Marlon Konertz, Fabian Dusse

**Affiliations:** 1 Department of Anesthesiology and Intensive Care Medicine, Faculty of Medicine and University Hospital University of Cologne, Cologne, Germany; 2 ProMEDITA gem. e.V. Registered Non-Profit Association, Essen, Germany; 3 Department of Anesthesiology and Intensive Care Medicine, University Witten/Herdecke, Cologne, Germany; 4 Institute of Medical Statistics and Bioinformatics (IMSB), Faculty of Medicine, University of Cologne, Cologne, Germany; 5 Department of Anesthesiology, Critical Care Medicine and Pain Therapy, Kliniken Essen-Mitte, Essen, Germany

**Keywords:** apprehension, clinical scenarios, emergency medicine education, emergency procedures, self-assessment, uncertainty

## Abstract

**Background:**

Emergency physicians face high cognitive and emotional stress in acute care, especially in prehospital settings. Structured training is an essential component of emergency medicine education and may increase familiarity with high-pressure clinical situations. In Germany, an 80-hour standardized emergency physician course is mandatory for certification.

**Objective/design:**

This study evaluated the impact of this course on physicians’ self-perceived apprehension and uncertainty in acute emergency scenarios using a prospective pre–post design.

**Settings and participants:**

The study was conducted in five consecutive emergency courses. 162 physicians from different specializations and departments throughout Germany completed pre- and post-course questionnaires.

**Intervention:**

Participants completed the standardized 80 h course, which combines theoretical instruction, structured decision-making, team communication, and high-fidelity simulation.

**Analysis:**

The primary endpoint was the change in a single combined self-reported rating of apprehension and uncertainty, assessed using a 5-point Likert scale across 41 questionnaire items. Analyses of the predefined categories constituted secondary analyses, while analyses of the 41 individual items and subgroup analyses were exploratory.

**Results:**

A significant overall reduction in combined self-reported apprehension and uncertainty was observed. Improvements occurred across all seven predefined categories. The greatest reductions were found for intraosseous access and ocular emergencies. Pediatric and neonatal emergencies remained the most apprehension-provoking scenarios. Exploratory subgroup analyses provided no evidence of differences in improvement across medical specialties or according to board certification status, whereas participants with prior EMS experience showed a smaller improvement than those without prior EMS experience.

**Conclusion:**

Course participation was associated with lower immediate post-course self-reported apprehension/uncertainty scores.

## 1 Introduction

Emergency medicine is a cornerstone of modern healthcare, requiring physicians and paramedics to make rapid, high-stakes decisions under pressure. Training in acute care must therefore not only impart clinical skills but also address the psychological demands associated with emergency care without compromising patient safety (1, 2). In the prehospital setting, where decisions are often made without full diagnostic clarity, structured and realistic training may support increase familiarity and confidence in managing acute clinical scenarios (3).

While emergency medicine is internationally recognized as a diverse and rewarding field, previous studies have reported high workload, occupational stress, burnout, and trauma-related symptoms in selected emergency care populations, including junior physicians (4–9). Reported contributors to psychological stress include diagnostic uncertainty, fear of medical errors, organizational barriers, high workload, and limited clinical experience (10).

Specific emergencies, notably pediatric and obstetric cases, have been identified as particularly stressful for emergency physicians (11). Structured educational and simulation-based training may therefore be especially relevant for increasing familiarity with infrequently encountered and high-pressure clinical situations (12). In many countries, certification as an emergency physician requires several years of clinical experience, a hands-on training course, and a final examination. Practical and simulation courses play a central role, covering, amongst others, cardiopulmonary resuscitation (CPR), airway management, circulatory stabilization, mass-casualty incidents (MCI), and pediatric emergencies (13).

Beyond clinical competence, apprehension and uncertainty may influence how physicians experience and approach high-pressure clinical situations. Although these responses may be adaptive in some circumstances, excessive apprehension and uncertainty may interfere with cognitive processing and decision-making. Accordingly, emergency medicine training increasingly incorporates approaches aimed at improving familiarity and confidence in acute scenarios (14). In Germany, the standardized board-certified 80-hour emergency physician course (“Notarztkurs”) represents a central component of prehospital emergency medicine qualification. This course imparts theoretical knowledge across the full spectrum of acute care, emphasizes structured decision-making and team communication, and develops practical skills in dynamic, high-fidelity simulated scenarios.

Although previous studies have examined stress, confidence, and other self-reported outcomes in emergency care education, evidence on scenario-specific changes in self-perceived apprehension and uncertainty before and after structured training remains limited, particularly across heterogeneous physician cohorts from different medical specialties. The aim of this prospective study was therefore to assess immediate changes in combined self-perceived apprehension and uncertainty across 41 emergency scenarios before and after participation in the standardized German emergency physician course. Secondary analyses examined predefined clinical categories, while exploratory analyses assessed individual items and participant subgroups.

## 2 Materials & methods

### 2.1 Study design

This prospective, uncontrolled, single-group before-and-after educational study complied with the Declaration of Helsinki, was approved by the Ethics Committee of the University of Cologne (No: 19-1389) and registered in the German Clinical Trials Registry (ID: 19377) before participant enrollment, on 15 November 2019. Data were collected and analyzed anonymously.

### 2.2 Setting

The emergency physician course took place in Westerland, Sylt, Germany, and was organized by ProMedita gem. e.V. (non-profit organization, Essen, Germany). Surveys were conducted on day one before the course and on the last day after completion.

### 2.3 Course structure

Successful completion of a standardized board-certified 80-hour course is a prerequisite for working as a prehospital emergency physician in Germany. The curriculum comprises 40% lecture-based theoretical instruction covering all aspects of emergency medical care, 35% practical training using realistic training models, including airway management manikins and a realistic thoracic drainage model. There are also joint exercises with emergency medical and fire and rescue services as well as a MCI exercise. The remaining 25% consists of high-fidelity simulation training involving a range of emergency scenarios conducted in small groups. The curriculum and the relative proportions of lectures, practical training, and simulation remained unchanged throughout the study period. The teaching faculty also remained consistent, with no relevant changes to the course structure or curriculum.

### 2.4 Participants

A total of 403 physicians were invited to participate in the study. Participants were informed about the study and provided informed consent by voluntarily completing the survey.

Inclusion criteria:

Regular participation in the course

Completion of both surveys.

Exclusion criteria:

Incomplete participation

Participation in only one survey

Invalid identification code preventing linkage of the pre- to the post survey.

### 2.5 Questionnaire

The questionnaire comprised 41 items covering emergency medicine topics, skills, and procedures, based on the curriculum specified by the German Medical Association. It was developed by the principal investigator and subsequently reviewed by five experts in accordance with the training regulations for obtaining the additional qualification in Emergency Medicine. Given the close alignment of the questionnaire items with the content and competencies stipulated in the applicable training regulations, no separate pilot testing was performed.

To link pre- and post-course questionnaires while ensuring anonymity, participants generated a six-character code using:

3rd and 5th letters of mother's maiden name

3rd and 5th letters of father's first name

1st and 2nd letters of first/favorite animal.

A combined self-reported rating of apprehension and uncertainty was assessed using a five-point Likert scale:

“Please rate your personal level of apprehension and uncertainty regarding the following emergency situations:”

1—Not at all apprehensive or uncertain
2—Slightly apprehensive or uncertain
3—Moderately apprehensive or uncertain
4—Apprehensive or uncertain
5—Very apprehensive or uncertain

Topics included:

**General**: CPR, shock, cardiac emergencies, respiratory emergencies, gastrointestinal emergencies, metabolic emergencies, neurological emergencies, intoxication, suicide emergencies, traumatic brain injury (TBI), psychiatric emergencies, abdominal/thoracic trauma, extremity trauma, polytrauma, burn injury, hypothermia, chest tube insertion, drowning/diving emergencies, electrical emergencies, ear, nose, and throat (ENT) emergencies, ocular emergencies, urological emergencies, gynecological emergencies, childbirth, MCI, pediatric emergencies, and initial newborn care.
**Adult- and pediatric-specific procedures**: anesthesia, airway management, cricothyrotomy, peripheral intravenous access (PIV), and intraosseous access.

Additional procedure-specific items included adult fracture management, adult positioning, pediatric fracture management, and pediatric fracture immobilization.

### 2.6 Endpoints

The primary endpoint was the within-participant change from pre- to post-course in the mean self-reported apprehension and uncertainty score across all 41 items.

Analyses of the seven predefined categories were considered secondary analyses, whereas analyses of the 41 individual items and subgroup analyses were considered exploratory. Exploratory subgroup analyses were performed by specialty (anesthesiology, internal medicine, surgery, other), prior emergency medical service (EMS) experience, and board certification.

### 2.7 Statistical analysis

For the descriptive analysis, numerical variables are presented as means ± standard deviations, whereas categorical and dichotomous variables are reported as frequencies and proportions (%). Mean differences are reported as delta (*Δ*), together with corresponding 95% confidence intervals (CIs).

To provide an overall descriptive measure, the mean rating across all 41 items was calculated as a composite index of combined self-reported apprehension and uncertainty across the assessed emergency scenarios. The primary endpoint was the within-participant change from pre- to post-course in the mean self-reported apprehension and uncertainty score across all 41 items. The overall within-participant change in the mean score across all 41 items was defined as the primary analysis. For this analysis, the 41 item responses were first aggregated at the participant level to obtain one overall mean score per participant and time point; individual item responses were therefore not treated as independent observations. Within-participant dependence between pre- and post-course measurements was accounted for by the paired design. The distributions of the paired differences were assessed for substantial deviations from normality by visual inspection of Q–Q plots. For the primary paired pre–post comparison, the standardized effect size was calculated as Cohen's d_z_, defined as the mean within-participant difference divided by the standard deviation of the paired differences.

Analyses of the seven predefined categories were considered secondary analyses. To account for multiple testing, *p* values were adjusted using the Holm procedure separately within each family of hypotheses. Multiplicity adjustment was therefore performed separately for the seven category-level comparisons, the 41 item-level comparisons, the three subgroup comparisons and the seven between-specialty comparisons of change within the predefined categories. The primary analysis was not subject to multiplicity adjustment. All statistical tests were two-sided. A *p*-value <0.05 was considered statistically significant. For analyses subject to multiplicity adjustment, statistical inference was based on Holm-adjusted *p* values.

Subgroup analyses were considered exploratory. For each participant, change scores were calculated as the pre-course minus the post-course score. Differences in the overall change score across medical specialties were assessed using one-way ANOVA, whereas differences according to prior EMS experience and board certification status were assessed using independent-samples t-tests. The *p* values from these three subgroup analyses were adjusted jointly using the Holm procedure. In an additional exploratory analysis, between-specialty differences in change were assessed separately for each of the seven predefined clinical categories using one-way ANOVA. The resulting seven *p* values were adjusted jointly using the Holm procedure. Pairwise *post hoc* comparisons using Tukey's test were reported for categories with a significant Holm-adjusted test; no additional Holm adjustment was applied to these *post hoc* comparisons because multiplicity was already accounted for within the Tukey procedure. Subgroup results are reported as effect estimates with corresponding 95% confidence intervals.

Although individual questionnaire items were assessed on a five-point Likert scale, the primary endpoint was based on the mean score across all 41 items and was treated as an approximately continuous measure. Sensitivity analyses using Wilcoxon signed-rank tests were additionally performed for the primary overall score and the seven category-level scores.

All statistical analyses were performed using IBM SPSS Statistics, version 29.0 (IBM Corp., Armonk, NY, USA). Figures were created using Microsoft Excel and Microsoft PowerPoint (Microsoft Corp., Redmond, WA, USA).

The 41 questionnaire items were grouped into seven predefined clinical categories by the principal investigator in consultation with five experts in accordance with the applicable training regulations for the additional qualification in Emergency Medicine.

**Resuscitation** (CPR, shock)
**Internal Medicine** (cardiac, respiratory, gastrointestinal, and metabolic emergencies)
**Neurology and psychiatry** (neurological emergencies, intoxication, suicide emergencies, TBI, psychiatric emergencies)
**Trauma** (abdominal/thoracic trauma, extremity trauma, polytrauma, burn injury, hypothermia, adult fracture management, adult positioning, chest tube insertion)
**Special Emergencies** (drowning and diving emergencies, electrical emergencies, ENT emergencies, ophthalmological emergencies, urological emergencies, mass casualty incidents)
**Airway management and anesthesia** (adult and pediatric anesthesia, adult and pediatric airway management, adult and pediatric PIV, adult and pediatric intraosseous access, adult and pediatric cricothyrotomy)
**obstetrics/gynecology/pediatrics** (gynecological emergencies, childbirth, pediatric and neonatal emergencies, pediatric fracture management and immobilization)

## 3 Results

Data collection took place between 2019 and 2022. Datasets from 162 participants who completed pre- and post-questionnaires were analyzed. Details on specialty, prior EMS experience, and board certification status are shown in Figure 1. Participant enrollment details are shown in Figure 2. No duplicate identifiers or code collisions were observed in the study dataset.

**Figure 1:**
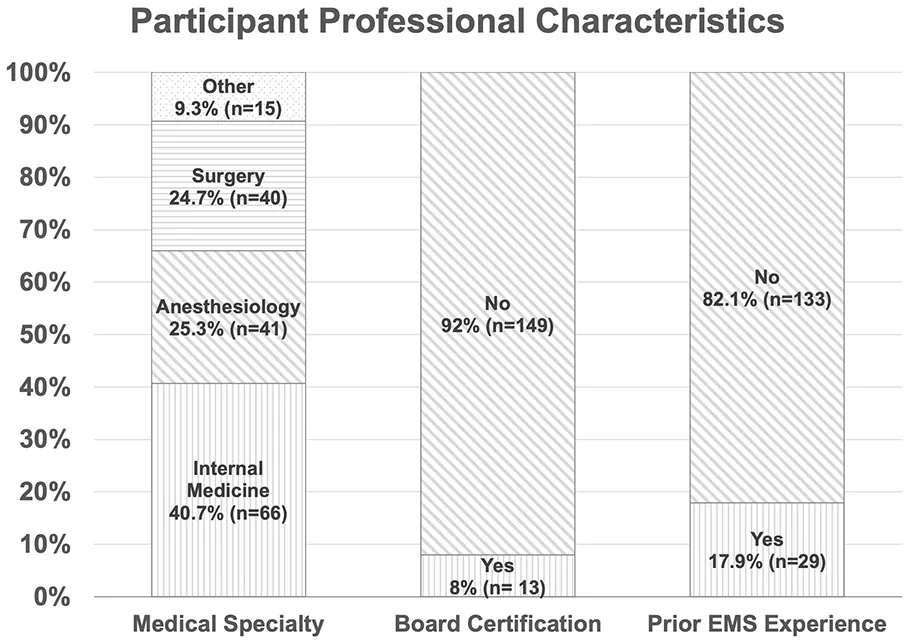
Participant professional characteristics: distribution of medical specialty, board certification status, and prior emergency medical service experience among all study participants (*n* = 162).

**Figure 2:**
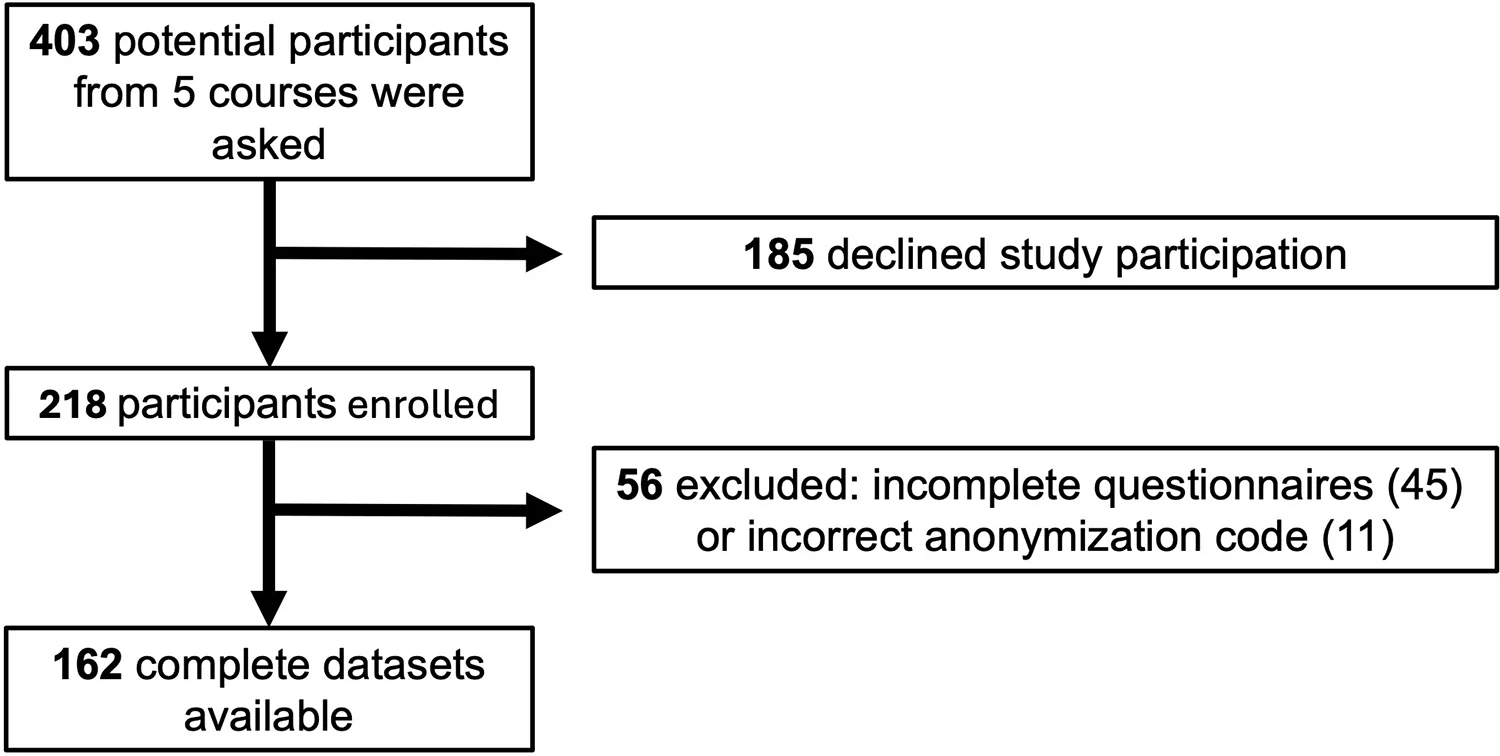
Flow chart of participant enrollment and dataset inclusion.

### 3.1 Pre–post analysis across all items

The mean score across all 41 items decreased from 3.31 ± 0.54 before the course to 2.60 ± 0.50 after the course. The mean within-participant reduction was 0.71 points (95% CI: 0.65–0.77; t(161) = 23.51; *p* < 0.001), corresponding to a standardized effect size of Cohen's d_z_ = 1.85. The sensitivity analyses using Wilcoxon signed-rank tests yielded results consistent with the primary parametric analyses, with significant pre–post differences for the overall score (*p* < 0.001).

### 3.2 Analysis by individual item

The highest pre-course scores were observed for pediatric cricothyrotomy (4.59), pediatric emergencies (4.42), and initial newborn care (4.38), whereas the lowest pre-course scores were observed for adult PIV (1.49), gastrointestinal emergencies (2.60), and adult anesthesia (2.62). The greatest improvements were observed for pediatric intraosseous access (*Δ*=1.25), adult intraosseous access (*Δ*=1.13), and ocular emergencies (*Δ*=1.02). The smallest improvements were observed for adult PIV (*Δ*=0.11), gastrointestinal emergencies (*Δ*=0.36), and urological emergencies (*Δ*=0.40) (Table 1).

**Table 1:** Pre- and post-course comparison of self-reported apprehension and uncertainty across 41 emergency medicine items before and after the course (mean values, standard deviations, 95% confidence intervals, and *p*-values).

Item	Pre mean vs. post mean; difference mean ± SD	95% CI	Holm adjusted *p* values	Item	Pre mean vs. post mean; difference mean ± SD	95% CI	Holm adjusted *p* values
CPR	2.63 vs. 1.91; 0.72 ± 0.89	0.58–0.86	<0.001	ENT emerg.	3.70 vs. 2.78; 0.92 ± 0.93	0.78–1.06	<0.001
Shock	2.94 vs. 2.30; 0.64 ± 0.80	0.52–0.77	<0.001	Ocular emerg.	3.60 vs. 2.59; 1.02 ± 0.92	0.88–1.16	<0.001
Cardiac emerg.	2.75 vs. 2.27; 0.48 ± 0.74	0.37–0.60	<0.001	Urological emerg.	3.02 vs. 2.62; 0.40 ± 0.82	0.27–0.53	<0.001
Respiratory emerg.	2.98 vs. 2.36; 0.61 ± 0.89	0.47–0.75	<0.001	MCI	4.21 vs. 3.43; 0.78 ± 0.97	0.63–0.93	<0.001
Gastrointestinal emerg.	2.60 vs. 2.23; 0.36 ± 0.75	0.25–0.48	<0.001	Adult anesthesia	2.62 vs. 2.19; 0.43 ± 0.81	0.31–0.56	<0.001
Metabolic emerg.	2.97 vs. 2.45; 0.52 ± 0.77	0.40–0.64	<0.001	Pediatric anesthesia	3.91 vs. 2.98; 0.94 ± 0.94	0.79–1.08	<0.001
Neurological emerg.	3.11 vs. 2.38; 0.74 ± 0.86	0.60–0.87	<0.001	Adult airway mgmt.	3.04 vs. 2.44; 0.60 ± 0.92	0.46–0.74	<0.001
Intoxication	3.20 vs. 2.62; 0.58 ± 0.79	0.46–0.70	<0.001	Pediatric airway mgmt.	4.10 vs. 3.12; 0.99 ± 0.98	0.84–1.14	<0.001
Suicide emerg.	3.07 vs. 2.60; 0.47 ± 0.87	0.33–0.60	<0.001	Adult cricothyrotomy	4.02 vs. 3.28; 0.75 ± 0.90	0.61–0.89	<0.001
TBI	3.26 vs. 2.47; 0.79 ± 0.82	0.66–0.95	<0.001	Pediatric cricothyrotomy	4.59 vs. 3.93; 0.66 ± 0.92	0.52–0.80	<0.001
Psychiatric emerg.	3.19 vs. 2.78; 0.41 ± 0.79	0.29–0.54	<0.001	Adult PIV	1.49 vs. 1.38; 0.11 ± 0.55	0.03–0.20	0.01
Abdominal/ thoracic trauma	3.35 vs. 2.56; 0.78 ± 0.86	0.66–0.93	<0.001	Pediatric PIV	3.27 vs. 2.73; 0.54 ± 1.14	0.37–0.72	<0.001
Extremity trauma	3.06 vs. 2.24; 0.82 ± 0.86	0.69–0.95	<0.001	Adult intraosseous access	2.69 vs. 1.56; 1.13 ± 0.90	0.99–1.27	<0.001
Polytrauma	3.65 vs. 2.79; 0.86 ± 0.79	0.74–0.98	<0.001	Pediatric intraosseous access	3.40 vs. 2.15; 1.25 ± 1.15	1.06–1.43	<0.001
Burn injury	3.29 vs. 2.60; 0.69 ± 0.87	0.55–0.82	<0.001	Gynecological emerg.	3.74 vs. 2.99; 0.75 ± 0.89	0.61–0.89	<0.001
Hypothermia	3.18 vs. 2.57; 0.61 ± 0.93	0.47–0.76	<0.001	Childbirth	4.20 vs. 3.27; 0.93 ± 0.84	0.80–1.06	<0.001
Adult fracture mgmt.	2.63 vs. 1.96; 0.67 ± 0.83	0.55–0.80	<0.001	Pediatric emerg.	4.42 vs. 3.44; 0.98 ± 0.87	0.84–1.11	<0.001
Adult Positioning	2.73 vs. 2.04; 0.69 ± 0.87	0.56–0.83	<0.001	Initial newborn care	4.38 vs. 3.43; 0.96 ± 1.01	0.80–1.11	<0.001
Chest tube insertion	3.22 vs. 2.41; 0.81 ± 0.96	0.66–0.96	<0.001	Pediatric fracture mgmt.	3.03 vs. 2.38; 0.65 ± 1.02	0.49–0.81	<0.001
Drowning/Diving emerg.	3.72 vs. 3.06; 0.66 ± 0.87	0.53–0.80	<0.001	Pediatric fracture immobilization	3.17 vs. 2.38; 0.80 ± 0.97	0.65–0.95	<0.001
Electrical emerg.	3.36 vs. 2.78; 0.58 ± 0.85	0.45–0.71	<0.001				

*P* values for the 41 exploratory item-level comparisons were adjusted for multiple testing using the Holm procedure.

CI, confidence interval; CPR, cardiopulmonary resuscitation; emerg., emergencies; ENT, Ear, Nose and Throat; mgmt., management; PIV, peripheral intravenous line; TBI, traumatic brain injury.

### 3.3 Category analysis

Significant pre–post improvements were observed across all seven predefined categories (Table 2/ Figure 3), with all differences remaining statistically significant after Holm adjustment for multiple testing (all adjusted *p* < 0.001). The sensitivity analyses using Wilcoxon signed-rank tests yielded results consistent with the corresponding parametric analyses, with significant pre–post differences across all seven category-level scores (all *p* < 0.001).

**Table 2:** Pre- and post-course apprehension and uncertainty toward seven emergency-category groups.

Category	Pre-course mean ± SD	Post-course mean ± SD	Mean difference ± SD	95% confidence interval	Cohen's d_z_	Holm adjusted *p* values
Resuscitation	2.78 ± 0.85	2.10 ± 0.68	0.68 ± 0.70	0.57–0.79	0.98	<.001
Internal	2.82 ± 0.78	2.33 ± 0.63	0.49 ± 0.55	0.41–0.58	0.90	<.001
Neuro	3.17 ± 0.70	2.57 ± 0.60	0.60 ± 0.50	0.52–0.68	1.19	<.001
Trauma	3.14 ± 0.83	2.40 ± 0.65	0.74 ± 0.54	0.66–0.83	1.38	<.001
Special	3.60 ± 0.67	2.88 ± 0.56	0.73 ± 0.56	0.64–0.81	1.29	<.001
Airway	3.31 ± 0.66	2.57 ± 0.60	0.74 ± 0.55	0.66–0.83	1.35	<.001
OB/GYN/Ped	3.82 ± 0.69	2.98 ± 0.71	0.84 ± 0.59	0.75–0.93	1.42	<.001

Values are mean (Likert scale 1—not at all apprehensive or uncertain, 5—very apprehensive or uncertain) ± standard deviation and confidence interval for each scenario group before (“Pre-course”) and after (“Post-course”) the training intervention. *P* values for the seven category-level comparisons were adjusted for multiple testing using the Holm procedure. OB/GYN/Ped = obstetrics/gynecology/pediatric.

**Figure 3:**
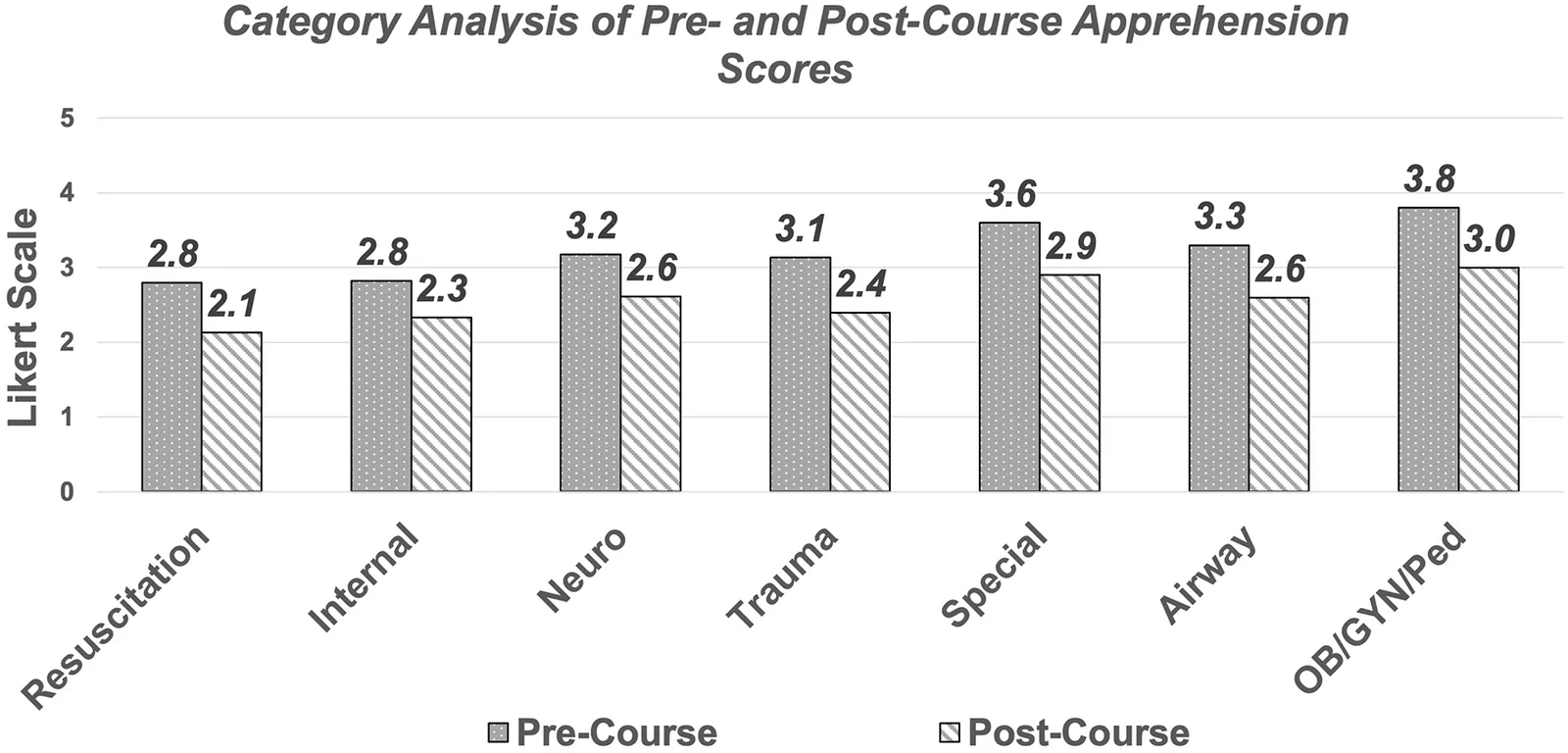
Average pre- and post-course Likert-scale apprehension and uncertainty ratings in emergency scenarios across seven emergency scenario groups. 1: Not at all apprehensive or uncertain. 2: Slightly apprehensive or uncertain. 3: Moderately apprehensive or uncertain. 4: Apprehensive or uncertain. 5: Very apprehensive or uncertain.

After Holm adjustment of the seven ANOVAs comparing changes between medical specialties, significant between-specialty differences remained for the categories Internal Medicine (adjusted *p* = 0.04) and Trauma (adjusted *p* = 0.003), whereas no significant differences were observed for the remaining five categories. Tukey-adjusted *post hoc* analyses showed greater improvement in the Internal Medicine category among surgical participants than among internal medicine participants (between-group *Δ*=0.37, 95% CI 0.06–0.69, *p* = 0.015). In the Trauma category, surgical participants showed smaller improvements than anesthesiologists (*Δ*=0.39, 95% CI 0.09–0.68, *p* = 0.005), internal medicine participants (*Δ*=0.39, 95% CI 0.13–0.66, *p* = 0.001), and participants from other specialties (*Δ*=0.48, 95% CI 0.08–0.89, *p* = 0.012).

### 3.4 Subgroup analyses

The magnitude of improvement in the overall mean score was similar across medical specialties (anesthesiology: *Δ*=0.69; surgery: *Δ*=0.70; internal medicine: *Δ*=0.72; other specialties: *Δ*=0.73), with no significant between-specialty difference in change (Holm-adjusted *p* = 0.969). Participants with prior EMS experience showed a smaller improvement than participants without prior EMS experience (*n* = 29, *Δ*=0.54 vs. *n* = 133, *Δ*=0.75; between-group difference in change: 0.21 points, 95% CI: 0.05–0.36; Holm-adjusted *p* = 0.026). There was no evidence of an association between board certification status and the magnitude of improvement (board-certified: *n* = 13, *Δ*=0.61; not board-certified: *n* = 149, *Δ*=0.72; between-group difference in change: 0.10 points, 95% CI: −0.12–0.32; Holm-adjusted *p* = 0.697).

## 4 Discussion

Working as an emergency physician involves repeated exposure to time-critical, high-stakes decision-making under unpredictable and stressful conditions. These situations are frequently unfamiliar, emotionally charged, and allow limited time for preparation. The resulting apprehension of the unknown and of being overwhelmed may impair clinical judgment and decision-making, compromising patient safety, and can also cause psychological distress among emergency physicians. In Germany, completion of a standardized board-certified 80-hour course is mandatory prior to certification as a prehospital emergency physician. This study evaluated changes in a combined self-reported rating of apprehension and uncertainty across 41 emergency scenarios before and after participation in this course among 162 physicians.

Emergency medicine in Germany is not recognized as an independent specialty, but rather is practiced by physicians from various disciplines, mostly, but not exclusively, anesthesiology, internal medicine, and surgery. Among participants, internal medicine was most frequent discipline, followed by anesthesiology, surgery, and others. This distribution appears to correspond approximately to the national distribution of medical specialties among physicians in Germany (15). An analysis of the German Trauma Registry reported that approximately 11,000 severely injured patients were primarily managed by anesthesiologists, followed by surgeons and internists (16). This discrepancy may be explained by the selected trauma population, the predominance of helicopter-based emergency care, and regional variation within the German emergency medical services system.

Previous studies examining stress and apprehension among emergency physicians, using subjective self-report measures or objective biomarkers such as cortisol levels, have identified several key stressors, including fear of medical errors, high workload, and structural or organizational challenges (4, 5, 10, 11, 17–20).

Spencer et al. reported an increased risk of psychological trauma among junior hospital staff who had witnessed an in-hospital cardiac arrest (9). This observation may be relevant to the present cohort, in which 37% of participants were second-year postgraduate trainees; however, vulnerability to stress-related effects was not directly assessed in our study.

In this study, the overall mean score, the seven predefined category scores, and the individual scenario scores decreased from pre- to post-course assessment. These findings reflect a reduction in the combined self-reported rating of apprehension and uncertainty following course participation. They are broadly consistent with previous reports of favorable self-reported outcome changes after structured educational interventions incorporating theoretical and simulation-based training. However, direct comparisons are limited due to differences in study populations, interventions, and outcome measures (19, 21–24). Moreover, current European guidelines emphasize the importance of high-quality education and ongoing training in emergency medicine as components of professional training and patient safety strategies (1, 25).

The results are also consistent with previous reports indicating that pediatric and obstetric emergencies are perceived as particularly challenging and stressful scenarios (11). In this study, pediatric emergencies, including general pediatrics, cricothyrotomy, and neonatal care, showed the highest levels of combined apprehension and uncertainty both before and after the course. Notably, “childbirth” ranked only in the upper quartile. This may reflect differences in perceived complexity, familiarity, or expected intervention requirements across obstetric scenarios; however, these factors were not directly assessed. Pediatric and obstetric emergencies have previously been reported as particularly stressful for emergency physicians, potentially because of their relative infrequency, limited routine exposure, and requirement for specific clinical expertise (26–28). Greater emotional burden has also been described in pediatric emergency care and may contribute to higher self-reported apprehension and uncertainty (29).

Exploratory subgroup analyses suggested specialty-specific differences in the magnitude of improvement in selected categories. Surgical participants showed smaller improvements in trauma-related scenarios than participants from the other specialty groups, while surgical participants showed greater improvement in the internal medicine category than internists. A similar pattern was observed for airway management/anesthesia, with smaller improvements among anesthesiologists; however, this between-specialty difference did not remain statistically significant after adjustment for multiple testing. Several mechanisms may account for these patterns. Greater baseline familiarity and specialty-specific experience may have resulted in lower pre-course apprehension and uncertainty, leaving less room for measurable reduction. Scale-related floor effects may therefore have contributed to the smaller observed changes in participants’ core areas of practice. Alternatively, the findings may reflect differences in prior experience or in the response to training. Conversely, larger reductions outside participants’ core specialties may primarily reflect greater room for change in self-reported ratings. Importantly, these findings reflect between-group differences in the magnitude of change and should not be interpreted as an absence of within-group improvement. Given the exploratory nature of these analyses, the findings should be interpreted cautiously.

Tapasak et al. reported subjective improvements in patient care and communication skills among medical students after an emergency medicine course (30). However, the absence of a pre-intervention assessment and the inclusion of medical students limit direct comparison with the present study. In contrast, the present study included 162 licensed physicians from multiple specialties and assessed changes across a broad range of emergency scenarios. These findings indicate a consistent reduction in participants’ perceived apprehension and uncertainty. However, clinical competence, preparedness, and patient-related outcomes were not directly assessed. Accordingly, these results should be regarded as complementary to, rather than directly comparable with, studies evaluating educational performance, communication skills, or clinical competence.

Justo et al. reported a significant reduction in perceived stress among 38 primary care physicians following a meditation-based intervention (31). Given the differences in intervention, study population, sample size, and outcome, direct comparison with the present findings is limited. Their study differed from the present study in terms of intervention, sample size, clinical population, and context. While their findings addressed perceived stress following a targeted psychological intervention, the present study examined changes in combined self-reported apprehension and uncertainty following a standardized emergency medicine course.

Similarly, O'Keeffe et al. demonstrated that a one-day course incorporating case-based discussions, active listening exercises, and simulation-based communication training was associated with improvements in participants’ non-technical skills, with 85% reporting enhanced conflict management abilities and 92% applying skills weekly (23). While their study underscores the potential value of structured, interactive training in improving specific interpersonal competencies, it focused primarily on communication and workplace interactions. In contrast, the present study assessed a standardized emergency medicine course in 162 physicians from multiple specialties and clinical affiliations. By evaluating changes in a combined self-reported rating of apprehension and uncertainty across 41 distinct emergency scenarios, the present findings provide additional evidence specifically regarding changes in these self-perceived responses following emergency medicine training.

Klein et al. demonstrated that simulation-based training targeting in-hospital cardiac arrest was associated with a significant reduction in anxiety symptoms at three-month follow-up (32). Their findings are not directly equivalent to the present results because they concerned a specific clinical scenario, a different psychological outcome, and a longer follow-up period. The present study instead examined immediate changes in self-reported apprehension and uncertainty following a broad emergency medicine course. In a cohort of physicians from various specialties and clinical backgrounds, reductions in combined self-reported apprehension and uncertainty were observed across 41 emergency scenarios. Whether these changes persist over time or are associated with objective clinical performance or patient-related outcomes remains to be determined.

## 5 Conclusion

Participation in the standardized German emergency medicine course was associated with a reduction in combined self-perceived apprehension and uncertainty across 41 emergency scenarios. Reductions were consistently observed across all seven predefined clinical categories and were comparable across medical specialties overall, although exploratory analyses identified specialty-specific differences in selected domains and smaller reductions among participants with prior EMS experience. Exploratory findings further suggested greater reductions in some areas outside participants’ routine clinical practice. As the study assessed self-perceived apprehension and uncertainty rather than objective clinical performance, the persistence and clinical relevance of these effects warrant further investigation.

Further research should assess the long-term persistence of these changes and whether periodic refresher training can sustain reductions in self-perceived apprehension and uncertainty. Examining the relationship between apprehension and uncertainty, perceived self-confidence, objective clinical competence, and patient-related outcomes may further clarify the clinical relevance of these self-reported changes.

### 5.1 Limitations

This study has several limitations. First, as a non-randomized observational study without a control group, causal relationships between course participation and reductions in perceived apprehension and uncertainty cannot be definitively established. The pre–post design is also susceptible to response-shift bias, as participation in the course may have altered participants’ understanding of emergency situations and their internal frame of reference for rating apprehension and uncertainty.

Second, self-reported Likert-scale ratings are subjective and may be influenced by social desirability, expectations of course benefit, and demand characteristics. The questionnaire was developed specifically for this study and has not undergone formal psychometric validation. Repeated administration may also have introduced recall or familiarity effects. Moreover, self-report measures do not objectively reflect actual clinical competence or behavior in emergency situations.

Third, the sample included only physicians who completed the mandatory emergency medical course between 2019 and 2022 and voluntarily participated in the survey, which may have introduced selection bias.

Fourth, follow-up was limited to the immediate post-course assessment; therefore, the persistence of the observed changes and their translation into clinical practice remain unclear.

Fifth, the primary endpoint combined apprehension and uncertainty into a single measure, preventing assessment of these constructs as independent outcomes.

Sixth, the 41 heterogeneous questionnaire items were aggregated into a composite index that should not be interpreted as a validated psychometric scale representing a single underlying construct and may mask scenario-specific differences. Although multiple testing was addressed using the Holm procedure for predefined secondary and exploratory analyses, the large number of item-level comparisons should be considered when interpreting these findings. Correlations between questionnaire items were not explicitly modeled in the item-level analyses; however, the primary analysis used participant-level aggregated scores and did not treat individual item responses as independent observations.

Seventh, exploratory subgroup analyses were limited by small and imbalanced group sizes, particularly for board-certified participants, participants with prior EMS experience, and the “Other” specialty group, resulting in reduced statistical power and precision. Therefore, non-significant findings should not be interpreted as evidence of absence of subgroup effects.

Eigth, course affiliation was not retained in the anonymized dataset, precluding course-specific analyses. Although course structure, curriculum, and teaching faculty remained consistent, between-course differences cannot be excluded.

Ninth, despite diverse clinical affiliations and specialties, generalizability beyond Germany's physician-staffed prehospital emergency system (the “Notarzt” model) may be limited because of differences in healthcare systems, training pathways, and emergency medical service organization.

Finally, persistently higher ratings for pediatric and neonatal scenarios may indicate areas warranting further educational attention but do not demonstrate inadequate course content, as they may also reflect the inherent complexity, rarity, or emotional demands of these scenarios.

## Data Availability

The analyzed data can be made available upon individual reasonable request, subject to applicable data protection regulations.
